# COVID-19 in Europe: from outbreak to vaccination

**DOI:** 10.1186/s12889-022-14454-5

**Published:** 2022-12-02

**Authors:** Paula Vicente, Abdul Suleman

**Affiliations:** Instituto Universitário de Lisboa (ISCTE-IUL), Business Research Unit (BRU-IUL), Lisboa, Portugal

**Keywords:** COVID-19, Pandemic, Vaccination, Fuzzy Analysis

## Abstract

**Background:**

COVID-19 is a pandemic of unprecedented proportions in recent human history. To date, the world has paid a high toll in terms of human lives lost, and on economic, financial, and social repercussions. In Europe, countries tried to mobilize all resources available to contain the COVID-19 effects, but the outcomes are diverse across countries. There have also been massive efforts geared towards finding safe and effective vaccines and to distribute them massively to the population. The main objective of this paper is to describe the COVID-19 prevalence in Europe. Secondly, it aims to identify epidemiological typologies allowing to distinguish the countries in terms of their response to the pandemic, and finally assess the effect of vaccination on pandemic control.

**Methods:**

The study covers 30 European countries: EU 27 in addition to Norway, Switzerland, and United Kingdom. Four epidemiological variables are analyzed at two distinct moments, at the end of 2020 and at the beginning of 2022: total number of cases per million, total number of deaths per million, total number of tests per thousand, and case fatality rate. In a second step, it uses a fuzzy approach, namely archetypal analysis, to identify epidemiological typologies, and positions countries by their response to the pandemic. Finally, it assesses how vaccination, stringency measures, booster doses and population age affect the case fatality rate, using a multiple regression model.

**Results:**

The outcomes unveil four epidemiological typologies for both periods. The clearest sign of change in the two periods concerns the case fatality rate that is found to be low in a single typology in 2020 but occurs in three typologies in 2022, although to different degrees. There is also statistical evidence of the positive impact of the primary vaccination on mortality reduction; however, the same does not hold for the booster dose and stringency measures.

**Conclusions:**

The study shows that primary vaccination is the most effective measure to reduce mortality by COVID-19 suggesting that vaccination provides hope for an end to the pandemic. However, a worldwide access to vaccination is needed to make this happen.

**Supplementary Information:**

The online version contains supplementary material available at 10.1186/s12889-022-14454-5.

## Introduction

COVID-19 is an infectious disease caused by the coronavirus SARS-CoV-2. It belongs to a large family of viruses that cause respiratory infections and, since its onset, has had a deleterious impact on the health of individuals worldwide. About $15\%$ of infected patients are severely affected and need oxygen and $5\%$ additionally present critical clinical conditions, requiring assistance in intensive care units. The odds of more serious symptoms are higher for elderly people as well as for those with comorbidities such as diabetes or heart disease [34].

**Fig. 1:**
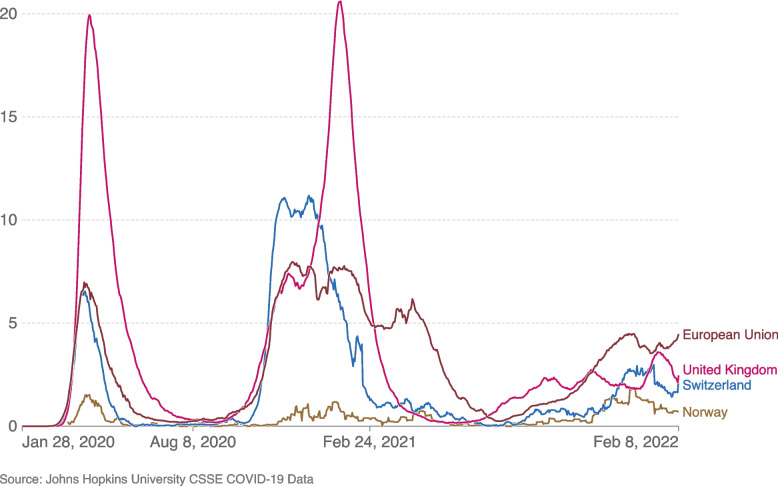
Daily new confirmed COVID-19 deaths per million people (7-day rolling average). Source: [22]

The COVID-19 outbreak epi-centered in Hubei Province of the People’s Republic of China in late December 2019, and rapidly spread to all over China and the world. By the end of January 2020, isolated cases appeared in some EU Member States. The first European case, with a travel history to China, was reported in France on 24 January 2020. On 30 January 2020 the World Health Organization (WHO) declared the outbreak of coronavirus to be a Public Health Emergency of International Concern and a pandemic on 11 March 2020. At the end of February 2020, Italy reported a significant increase in COVID-19 cases, mainly concentrated in the northern regions of the country, and by March 2020 all EU Member States had reported COVID-19 cases [12]. The first peak of deaths from COVID-19 in most European countries was registered in April 2020 (Fig. 1).

During the outbreak, many European Union/European Economic Area countries implemented both pharmaceutical and non–pharmaceutical interventions to contain the spread of the virus and the effects of the pandemic. Pharmaceutical measures include the use of face coverings outside of the home, testing and contact tracing. But the implementation of these measures was not uniform across countries and changed over the pandemic period. The use of face covering ranged from “No policy” to “Required outside the home at all times regardless of location or presence of other people”. Testing and contact tracing ranged from “No testing policy” (mostly in the initial months of the outbreak), to “Open public testing” at present [24]. Concomitantly, non-pharmaceutical measures were adopted: stay–at–home restrictions (orders–enforced, recommendations for the general population, recommendations for risk groups), closure of schools and workplaces (secondary schools/higher education, primary schools, daycare), cancellation of public events and gatherings, and international and domestic travel restrictions [24]. The first cycle of confinement extended roughly until the beginning of May 2020, and the restrictions were then relaxed gradually. By January-February 2021 another peak of deaths occurred (Fig. 1).

**Fig. 2:**
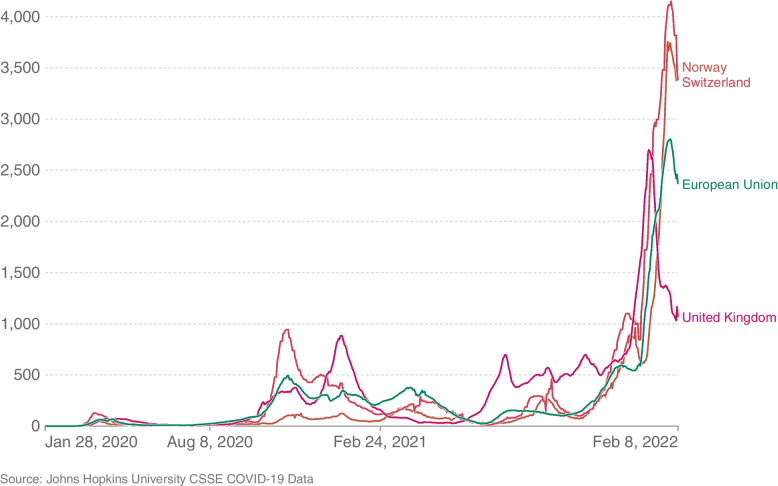
Daily new confirmed COVID-19 cases per million people (7-day rolling average). Source: [21]

All viruses change or mutate over time and SARS-CoV-2 is by no means an exception. This can lead to what is known as a variant to the main virus strain, which generally occurs where there is a large amount of community transmission. During late 2020, the emergence of variants that posed an increased risk to global public health prompted the characterization of specific variants of interest and variants of concern, to prioritize global monitoring and research, and ultimately to inform the ongoing response to the COVID-19 pandemic. The WHO has identified the variants of concern as Alpha, Beta, Delta, Gamma Omicron, first found in the United Kingdom, South Africa, India, Brazil and South Africa again, respectively. At the beginning of 2022, the Omicron variant became dominant in most European countries [25]. The Omicron variant spreads more easily than the original virus that causes both COVID-19 and the Delta variant. The European Centre for Disease Prevention and Control [11] confirms that anyone with the Omicron infection can spread the virus to others, even if they are vaccinated or asymptomatic. The dominance of this variant is to some extent responsible for the increase in the number of COVID-19 cases after December 2021 (Fig. 2).

Since the beginning of the outbreak, scientists have been working to develop and produce vaccines that could stop the spread of COVID-19. Prior to the COVID-19 pandemic, an established body of knowledge existed about the structure and function of coronaviruses causing diseases like severe acute respiratory syndrome (SARS) and Middle East respiratory syndrome (MERS). This knowledge boosted the development of various vaccine platforms and, on 8 December 2020, the first dose of the Pfizer/BioNTech vaccine was administered in the UK [4]. Approved COVID-19 vaccines by WHO (e.g. AstraZeneca/Oxford, Moderna, Pfizer/BioNTech) provide a high degree of protection against getting seriously ill and dying from the disease, although no vaccine is $100\%$ protective.

Many countries have implemented phased distribution plans that prioritize the vaccination of those at highest risk of complications, such as the elderly, and those at high risk of exposure and transmission, such as healthcare workers. However, COVID-19 vaccines are per se not enough to end this global health crisis unless everyone in the world has access to them. To this end, COVAX was launched in April 2020 as one of three pillars of the Access to COVID-19 Tools (ACT) Accelerator. Bringing together governments, global health organizations, manufacturers, scientists, private sector, civil society, and philanthropy, COVAX aims to provide innovative and equitable access to COVID-19 diagnostics, treatments, and vaccines, with special focus on the latter [37]. Millions of lives have been affected by travel restrictions, lockdowns and other measures put in place to reduce the spread of the virus. Millions have lost their jobs as the global economy slows. Health systems have become overwhelmed, making it difficult for patients with illnesses unrelated to COVID-19 to access treatment. WHO wants the vaccines provided by COVAX to contribute to reversing these trends and returning to normality. However, as of 7 January 2022, only $9\%$ of people in low-income countries had received at least one vaccine dose while in high-income states that percentage reaches $77\%$ [17]. In a highly interconnected global economy, effective and widely available vaccines are the fastest way to end the pandemic, restart the global economy and ensure a sustainable recovery but there is still a long way to go before we restore normality. The WHO continues to closely monitor the worldwide response by producing regular reports, guidelines, information, and registering relevant data regarding the number of infected and deaths, as well as the number of tests and vaccination. Since March 2022, the pandemic was slowing down in Europe [13, 21] but it is likely to rise again as deconfinement advances, the cold season returns and new variants emerge.

The main aim of this paper is to describe the COVID–19 prevalence in European countries at two distinct moments in time, namely by identifying epidemiological typologies and associating them with vaccination, primary and booster, with governments’ response stringency measures and with population age. Given the framework of a high contamination outbreak that was accelerated by the mutation rate of the virus, and considering the most reliable data available, four variables were selected to characterize the epidemiological impact of the virus on populations and health systems: Total number of cases per million (TNC); Total number of deaths per million (TND); Total number of tests per thousand (TT) and Case fatality rate (CFR). Specifically, the paper addresses the following research questions:

1. One year after the beginning of the outbreak (December 2020) how were European countries positioned in terms of TNC, TND, TT and CFR?
2. About one year after the beginning of vaccination (February 2022), how had the position of European countries changed regarding TNC, TND, TT and CFR?
3. What was the impact of vaccination and stringency measures on the CFR in European countries?
4. To what extent did the percentage of population aged 65+ affect the CFR of European countries?

## Methods

### Data

This study covers 30 countries, the 27 members of EU and three OECD surrounding countries, namely Norway, Switzerland, and United Kingdom (see [29] for a related work). For each one, epidemiological data were drawn from Our World in Data (OWD) [26] and relate to two distinct dates: 31 December 2020, i.e., about one year after the beginning of the pandemic, and 8 February 2022, about one year after the start of the vaccination program. The following variables are considered for statistical analysis: Total number of cases per million, Total number of deaths per million, Total number of tests per thousand, Case fatality rate (i.e., the ratio between the number of deaths among confirmed cases). For all these variables, cumulative values were collected regarding the two above mentioned dates. Information on vaccination was also retrieved from the OWD site and includes People fully vaccinated (two doses) per hundred and Total number of vaccine booster doses administered, divided by the total population of the country, both cumulative values on 8 February 2022. Booster doses are doses administered in addition to those prescribed by the original vaccination protocol [35]. Data on the percentage of population aged 65+ and a government stringency index were also retrieved. This index is a composite measure based on nine response indicators including school closures, workplace closure, cancellation of public events, restrictions on public gatherings, closures of public transport, stay-at-home requirements, public information campaigns, restrictions on internal movements, and international travel controls. The index is rescaled to a value from 0 to 100, where 100 means strictest response. The index is computed daily, and the data retrieved allowed us to compute an average value for the index^1^.

Although the OWD organization is a reliable source of statistical information on several domains, the gathering of accurate data during a pandemic crisis is not devoid of problems. It is consensual among statisticians that the estimation of the epidemiological models, for the purpose of making health decisions, has often been based on low amount or incomplete data [1]. The frequency of collecting and reporting data is not uniform across countries nor is the way COVID-19 deaths are counted. Even superficially similar places can have varying approaches to recording COVID-19 deaths. Early in the pandemic, countries such as the Netherlands counted only those individuals who died in hospital after testing positive for the coronavirus SARS-CoV-2. Neighboring Belgium included deaths in the community and everyone who died after showing symptoms of the disease, even if they were not diagnosed [1]. Most countries record every death and its cause, providing a permanent legal record. The health professional who records the death must note the primary cause of death and any contributing factors on the death certificate. And therein lies the problem. COVID-19 can lead to a greater chance of developing or aggravating other life threatening diseases, such as pneumonia, respiratory failure, blood clots, stroke, and heart attack. Furthermore, most of those who die after contracting COVID-19 have one or more comorbidities. This raises the question of how many people have died *of* COVID-19, and how many have died *with* COVID-19. Researchers in infectious diseases agree that the actual cause of death can be hard to determine. “Deaths are, to a degree, imprecise. A physician must make a judgment of cause of death.” Whether a person died of COVID-19 or with COVID-19 is open to interpretation and leads some to dispute official figures [16]. In an effort to make the reports of COVID-19 deaths consistent across countries, the WHO launched in April 2020 guidelines for certifying COVID-19 as a cause of death establishing that “A death due to COVID-19 is [...] a death resulting from a clinically compatible illness, in a probable or confirmed COVID-19 case, unless there is a clear alternative cause of death that cannot be related to COVID disease (e.g. trauma). There should be no period of complete recovery from COVID-19 between illness and death.” ([32], p.3).

### Statistical Analysis

The data analysis starts with a univariate statistical analysis of the epidemiologic variables performed using location and dispersion statistics, quantiles, and a box-plot analysis to visualize the empirical distribution of each variable and identify lowest and highest behaviors, namely possible outliers. The second step of our statistical analysis consists of verifying the most relevant profiles according to the above mentioned four epidemiological variables and seeing how countries changed during a 14-month period. We were particularly interested in profiles that were somehow extreme and, consequently, could help highlight the heterogeneity among countries. In sequel, we assume that the data hide a set of unknown $c\ge 2$ epidemiological typologies in each period and aim to verify how all countries are positioned in the structure set out by those typologies. This strategy, referred to as fuzzy analysis, allows partial membership and therefore makes it possible to account for how *c* typologies are shared by each country. In other words, the membership becomes a matter of degree and it is quantified by a number between 0 and 1, where 1 means full membership in the classical sense. This approach potentially provides a richer data analysis than a classical clustering which ends up with a crisp assignment, i.e. either belong or do not belong to a set, 1 or 0, respectively^2^.

For estimation purposes, we opted for an archetypal analysis [7] since it explores the extremal properties of data rather than their central properties as with the traditional fuzzy *c*-means [5] algorithm [19]. Archetypal analysis (AA) fits in the framework of the matrix factorization approach to data analysis. Here, it is assumed that the data matrix $\mathbf {X}=\left[ \mathbf {x}_{1}\ \mathbf {x}_{2}\ ...\ \mathbf {x}_{N}\right] =\left[ x_{jk}\right]$ $\in \mathbb {R}^{n\times N}$, where $n\ge 2$ is the dimension of the feature space, and $N>n$ is the sample size, can be approximated by a product of two matrices, $\mathbf {V}=\left[ v_{ji}\right] \in \mathbb {R}^{n\times c}$, $c\ge 2$, and $\mathbf {U}=\left[ \mu _{ik}\right] \in \left[ 0,1\right] ^{c\times N}$, such that $\sum _{i=1}^{c}\mu _{ik}=1,1\le k\le N$, i.e.

1 $$\begin{aligned} \mathbf {X\simeq VU,} \end{aligned}$$

the matrix of prototypes, $\mathbf {V}$, and the partition matrix $\mathbf {U}$. The $\mathbf {V}$ matrix configures a polytope with *c* extreme points, spanned by its *c* columns, namely $\mathbf {v}_{1}$, $\mathbf {v}_{2}$, ..., and $\mathbf {v}_{c}$. Each entry of the partition matrix $\mathbf {U}$, i.e. $\mu _{ik}$, is called membership degree and expresses the proportion of $\mathbf {v}_{i}$ present in $\mathbf {x}_{k}$. Therefore, every data point $\mathbf {x}_{k}$ is in the convex hull of *c* prototypes, apart from an error:

2 $$\begin{aligned} \mathbf {x}_{k}\mathbf {\simeq }\sum \limits _{i=1}^{c}\mu _{ik}\mathbf {v}_{i}. \end{aligned}$$

In the context of the present study, $\mu _{ik}$ can be fruitfully read as the proportion of typology *i* in country *k* or, equivalently, the amount of typology *i* shared by *k*.

AA is a special case of (1) where the prototypes are themselves convex combinations of data points,

3 $$\begin{aligned} \mathbf {v}_{i}=\sum \limits _{k=1}^{N}\beta _{ki}\mathbf {x}_{k},\ i=1,2,...,c, \end{aligned}$$

where $0\le \beta _{ki}\le 1$ and $\sum _{k=1}^{N}\beta _{ki}=1$. This restriction places prototypes within the data space and makes them archetypes [7]. In this way, the archetypes closely resemble certain data points and can therefore provide meaningful interpretation [3]. The (3) can also be written in matricial form as

4 $$\begin{aligned} \mathbf {V=XB}, \end{aligned}$$

where $\mathbf {B=}\left[ \beta _{ki}\right]$. Therefore, in AA the estimation of $\mathbf {V}$ is transferred to the estimation of $\mathbf {B}$ matrix.

Given a pre-specified value of *c*, the matrices $\mathbf {U}$ and $\mathbf {B}$ are often estimated by the minimization of the objective function

5 $$\begin{aligned} J_{c}\equiv J_{c}\left( \mathbf {U,V|X}\right) =\left\| \mathbf {X}-\mathbf {XBU}\right\| _{F}^{2}, \end{aligned}$$

subject to the constraints on $\mu _{ik}$ referred to above. The symbol $\left\| \mathbf {A}\right\| _{F}$ denotes the Frobenius norm of the matrix $\mathbf {A}$. Even though the objective function $J_{c}$ (5) is not convex in the product $\mathbf {BU}$, it is however convex in $\mathbf {U}$ and $\mathbf {B}$ separately. Therefore, an alternating optimization scheme is the most common procedure used for estimation purposes. In this study, we used the method provided by Bauckhage et al. in [3], which seems to be more efficient than some known alternatives (e.g. [2, 7, 18]). The goodness-of-fit was assessed using the validation index provided in [30], which relies on information-theoretic principles. We tested the models for $c=2,3,..,7$ archetypes, and selected the one that minimized the index. We note that the sample size is $N=30$, and 7 is sligthly higher than $\sqrt{N}$, which is a consensual upper bound for the number of clusters in data.

The final stage of our data analyses involves the estimation of a multiple linear regression (MLR) model to assess the determinants of the case fatality rate (CFR) in 2022. Specifically, we evaluate the impact of the following factors:

- Primary vaccination (VAC), measured by the percentage of people fully vaccinated per hundred;
- Age (AGE65), measured by the percentage of people aged 65 or plus in the country;
- Booster dose (BD), (i.e., doses administered beyond those prescribed by the original vaccination protocol), measured by the total number of vaccine booster doses administered, divided by the total population of the country;
- Stringency (ST), measured by an index ranging from 0 to 100, where 100 corresponds to the strictest response.

The theoretical MLR model to be estimated is defined as follows:

6 $$\begin{aligned} \text {CFR}=B_{0}+B_{1}\times \text {VAC}+B_{2}\times \text {AGE65}+B_{3}\times \text {BD}+B_{4}\times \text {ST}+\varepsilon \end{aligned}$$

where $\varepsilon$ is the error term, by assumption, normally distributed with 0 mean and standard deviation $\sigma$.

## Results

### Descriptive Statistics

We performed a univariate statistical analysis of the four quantitative epidemiological variables and present the results in Table 1. Complementarilly, the box-plot analysis provides a visualization of the empirical distribution of each variable. We realized that, even excluding the smallest and the highest observations (respectively, below first quartile and above third quartile), almost all the variables have a low relative dispersion as assessed by the Quartile Coefficient of Variation (QCV). We note that this statistic is a robust alternative to the coefficient of variation [6].

**Table 1:** Univariate analysis of the epidemiological variables for 30 countries in 2020 and 2022

Variable	Mean	SD	Q1	Median	Q3	QCV
in 2020						
TNC	36729.2	16483.3	24040.7	35719.5	50708.2	35.7
TND	714.2	399.6	400.1	683.6	1012.1	43.3
TT	587.5	498.9	353.7	460.6	596.3	25.5
CFR	1.97	0.87	1.40	1.80	2.53	28.8
in 2022						
TNC	237500.2	73340.6	170172.1	244050.2	290188.8	26.1
TND	2152.5	1122.0	1374.4	2007.7	2904.6	35.8
TT	4319.6	5304.8	1462.9	2425.9	4929.2	54.2
CFR	1.03	0.74	0.54	0.85	1.20	37.9

By $31^{st}$ December 2020 the total number of deaths is the most heterogeneous indicator across the countries under study, where QCV is $43.3\%$. By February 2022, it is the total number of tests that presents the highest heterogeneity ($54.2\%$), probably justified by different policies of testing adopted across countries during the second year of the pandemic.

**Fig. 3:**
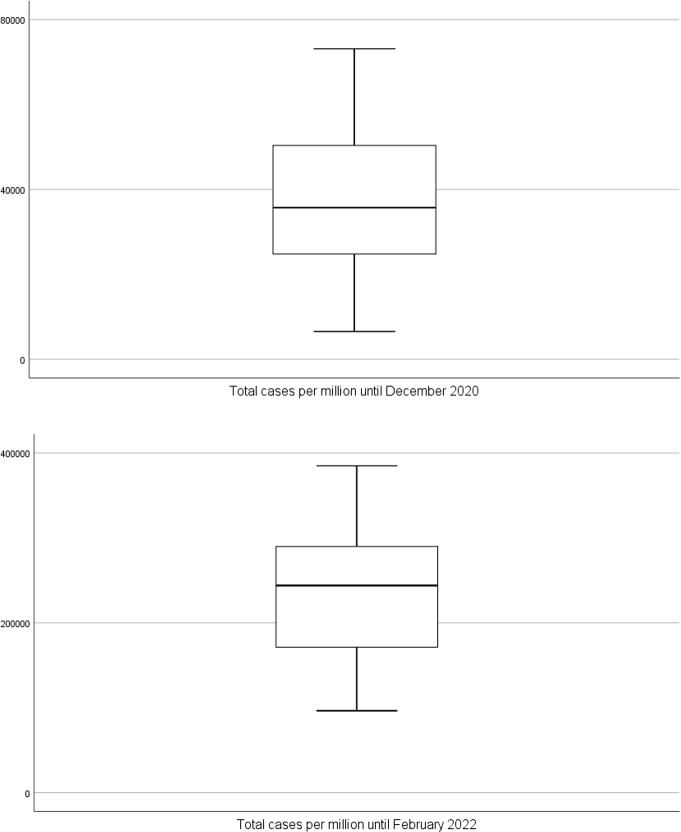
Box-plot for total number of cases until December 2020 and until February 2022 ($N=30$ countries)

Regarding the total number of COVID–19 cases until $31^{st}$ December 2020, Estonia, Finland, Germany, Greece, Ireland, Latvia, and Norway registered fewer than 24040.7 cases per million inhabitants (Q1) while Belgium, Croatia, Czech Republic, Lithuania, Luxembourg, Slovakia, Slovenia, and Switzerland reported more than 50708.2 cases per million inhabitants (Q3). By $8^{th}$ February 2022, the countries that reported fewest cases per million (below Q1) were Bulgaria, Finland, Germany, Hungary, Malta, Norway, Poland, and Romania, while Cyprus, Czech Republic, Denmark, France, Slovakia, and Slovenia reported more than 290188.8 cases per million inhabitants. The distribution of the total number of cases changed from positively skewed in 2020 to negatively skewed in 2022, which is a natural consequence of the increased spread of the virus (Fig. 3).

**Fig. 4:**
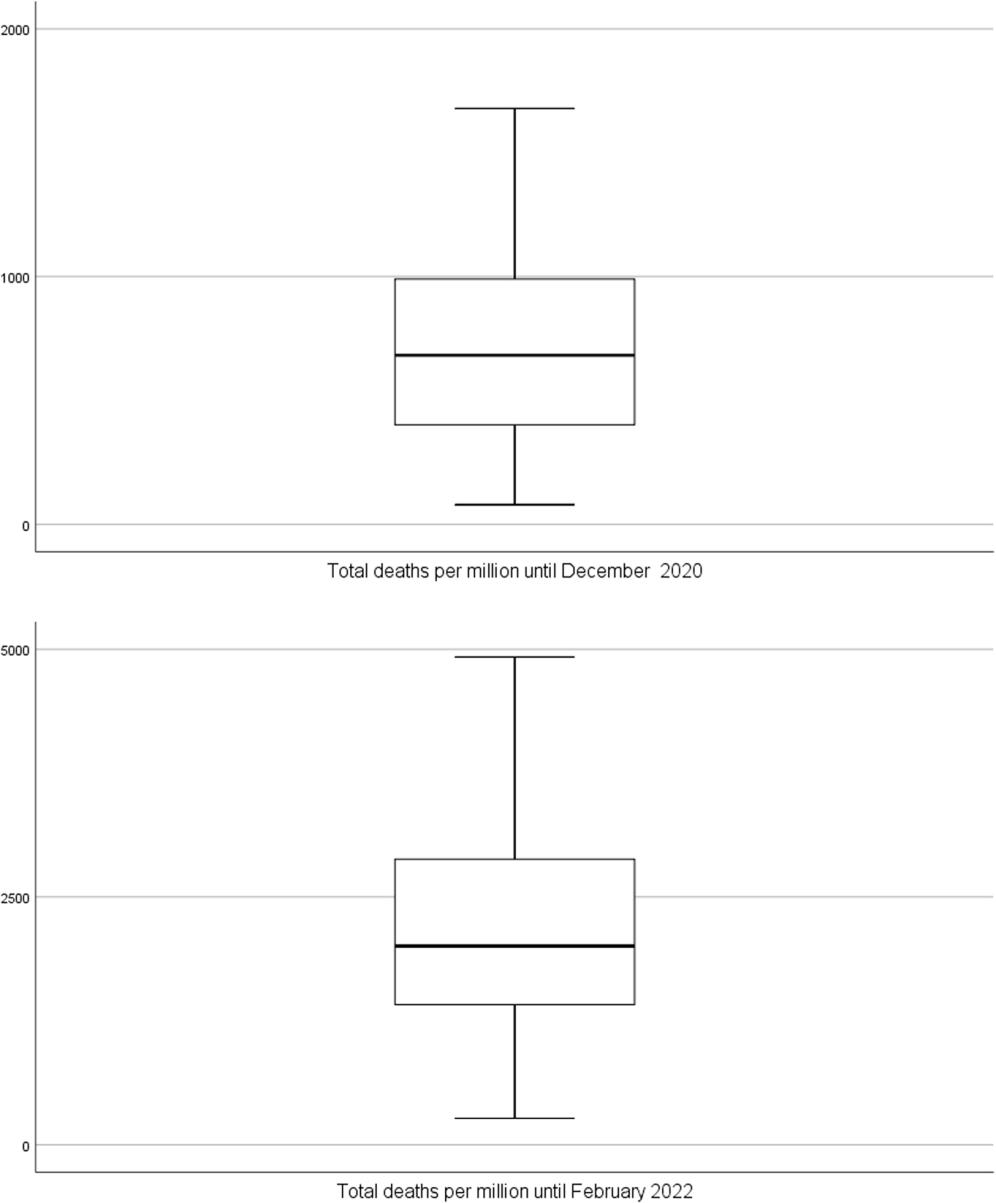
Box-plot for total number of deaths until December 2020 and until February 2022 ($N=30$ countries)

Regarding the number of COVID–19 deaths, Cyprus, Denmark, Estonia, Finland, Latvia, Norway, and Slovakia recorded fewer than 400.1 deaths per million until 31 December 2020 in contrast to Belgium, Bulgaria, Czech Republic, Italy, Slovenia, Spain, and United Kingdom with more than 1012.1 deaths per million inhabitants. By $8^{th}$ February 2022, Cyprus, Denmark, Finland, Ireland, Malta, Netherlands, and Norway recorded fewer than 1374.4 deaths per million while the highest number of deaths per million inhabitants (above Q3) were registered by Bulgaria, Croatia, Czech Republic, Hungary, Lithuania, Romania, and Slovakia (Fig. 4).

Mass COVID-19 diagnostic testing is a pharmaceutical strategy to control the spread of the SARS-CoV-2 which is achieved by: i) testing all contacts who have had high-risk exposure to COVID-19 cases, whether or not they are symptomatic, as soon as possible after they have been identified, to allow for further contact tracing; ii) testing all contacts who have had low-risk exposure to COVID-19 cases in settings where transmission is likely and/or the population is vulnerable to severe COVID-19; and iii) testing all contacts that become symptomatic [10]. The aim of identifying and managing the contacts of COVID-19 cases is to support early diagnosis and interrupt onward transmission by rapidly identifying and managing any secondary cases that may arise following transmission from primary cases. This is a less disruptive management strategy than non-pharmaceutical measures (e.g., stay-at-home orders and shutdowns of non-essential businesses), which are socially more costly tools to control the pandemic spread of SARS-CoV-2. Howerton et al. [15] provide evidence that increasing testing capacity, including the number of tests available and the speed at which test results are provided, can reduce reliance on costly preventative interventions.

**Fig. 5:**
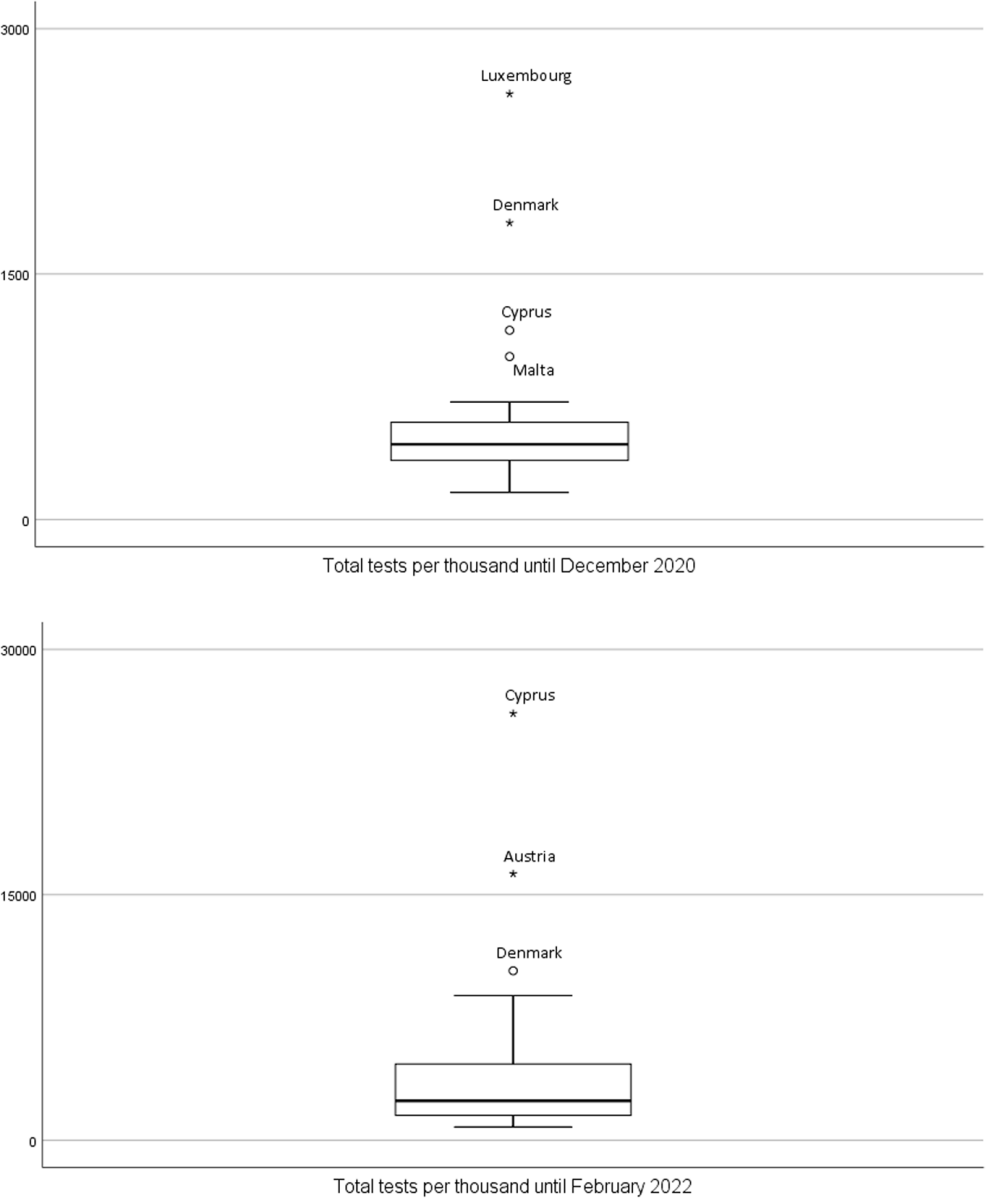
Box-plot for total number of tests until December 2020 and until February 2022 ($N=30$ countries)

The distribution of the total tests per thousand is particularly asymmetric with Luxembourg, Denmark, Cyprus, and Malta appearing as outlier countries in 2020 (with more than 997 tests per thousand inhabitants) and Cyprus, Austria and Denmark appearing as outlier countries in 2022, with more than 10369 tests per thousand inhabitants (Fig. 5).

The varying test strategies adopted by different countries contribute to different outcomes in terms of tests per thousand. Austrians can easily purchase saliva rapid tests in drug stores to be used at home. The kits can then be dropped at supermarkets, schools and even gas stations for processing, making the whole endeavor far less staff-intensive on all fronts. Moreover, lab costs are probably considerably lower in Austria. That is due to the way that specimens are processed. In Vienna, tests are pooled by Lifebrain, a leading European laboratory operator. That means the contents of 10 kits are robotically tested as a single batch. If the batch is positive, the tests are then analyzed individually. That saves time and money as Lifebrain only charges a few euros to process a single PCR test [14]. In Denmark, a country of only 5.8 million, the number of COVID-19 tests being carried out is also high. A lateral flow test can easily be done in one of over 400 test centers without an appointment. In case of a PCR test, people can register via a national website. Even when demand is high, laboratories are expected to return at least $80\%$ of PCR test results by the following day. Lateral flow tests, as well as PCR tests, cost nothing in Denmark, in contrast with other countries (e.g., in Germany, PCR tests range in price from about €$50-$€70 and in Portugal from about 90€$-120$€, for people not belonging to a high priority group). Denmark has been a testing trailblazer since the start of the pandemic, carrying out some 117 million tests, half PCR type and the other half lateral flow tests [14].

**Fig. 6:**
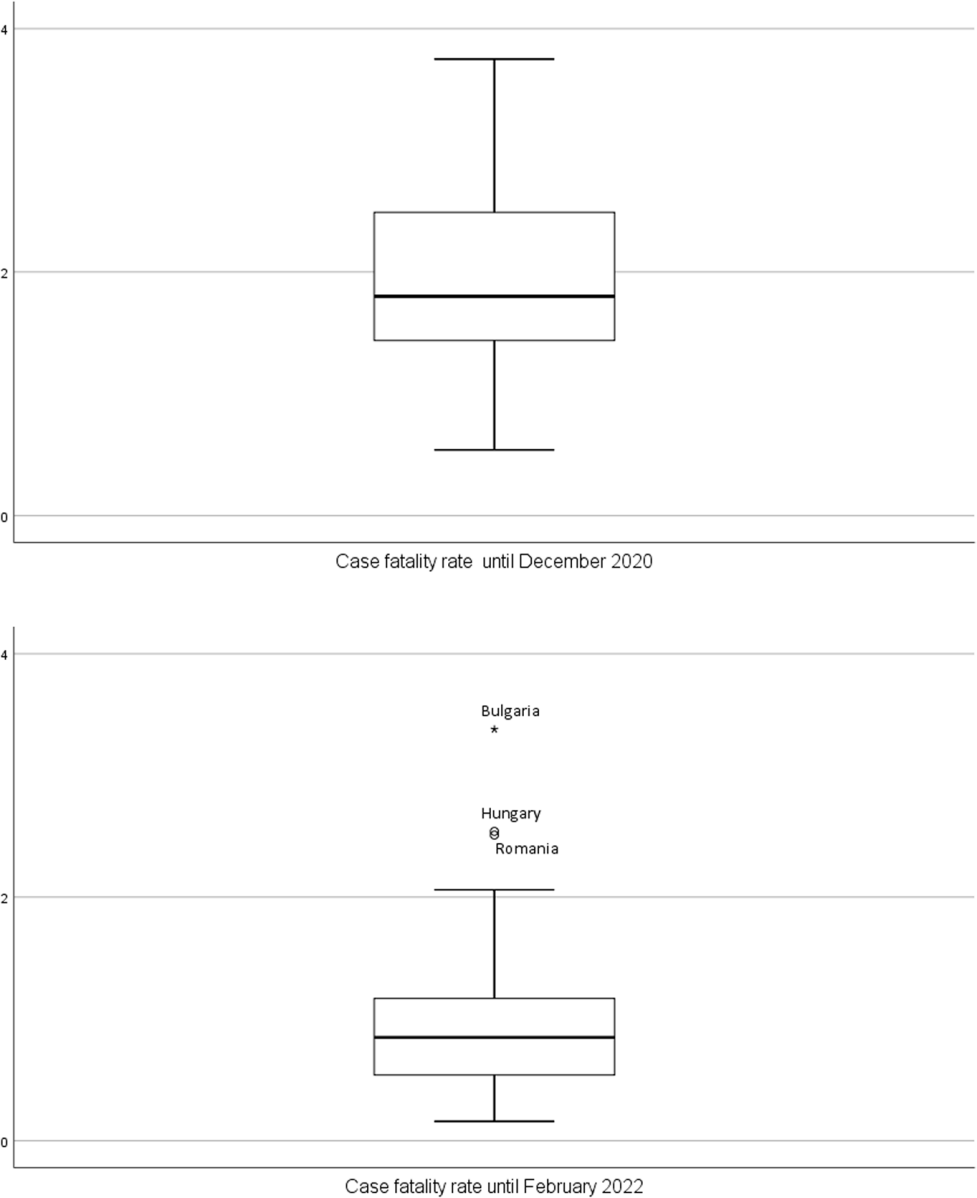
Box-plot for case fatality rate until December 2020 and until February 2022 ($N=30$ countries)

As for the case fatality rate, Cyprus, Denmark, Estonia, Lithuania, Luxembourg, Norway, and Slovakia recorded a case fatality rate below $1.4\%$ (Q1) in December 2020, while Bulgaria and Hungary recorded the highest case fatality rates (above $2.53\%$) in that date. The distribution of this variable becomes strongly asymmetric in 2022, with outlier countries appearing with case fatality rates above $2.06\%$ for Bulgaria, Hungary, and Romania (Fig. 6). To better understand these figures it is important to note that CFR is the ratio between the number of confirmed deaths from COVID-19 and the number of confirmed cases, not total cases since many cases are not confirmed. Moreover, the probability that someone dies of COVID-19 does not depend just on the disease itself, but also on the treatment applied and the patient’s own recovering ability. This means that the CFR can decrease or increase over time, as responses change; and it can vary by location and by the characteristics of the infected population, such as age, or sex [23]. The overall decrease in CFR from 2020 to 2022 is likely the consequence of most pharmaceutical and non-pharmaceutical measures undertaken by the countries during the pandemic period (the median in 2020 and 2022 are $1.8\%$ and $0.85\%$, respectively).

### Epidemiological Typologies

For the decomposition purposes by means of an AA, we used the standardized version of the data, since the variables underlying the analysis, i.e. total number of cases per million, total number of deaths per million, total number of tests per thousand and case fatality rate, have different magnitudes as well as different measurement scales. However, for a better substantive interpretation, the results are presented in their original units.

**Table 2:** Goodness-of-fit index for archetypal analysis of 2020 and 2022 data.

Archetypes	Index Value	
*c*	2020	2022
2	6.00	6.11
3	4.07	4.26
4	2.77	1.19
5	2.87	2.23
6	3.35	2.12
7	4.34	2.85

Table 2 presents the goodness-of-fit index for $c=2,3,..,7$ typologies when performing archetypal analysis for 2020 and 2022 data. The solution of $c=4$ archetypes is optimal for both datasets, 2020 and 2022, as indicated by the minimum value of the index. Therefore, our analysis will be explored from a perspective of $c=4$ epidemiological typologies. Table 3 shows how typologies are characterized according to the epidemiological variables. The typologies are displayed in increasing order of case fatality rate (CFR). Since CFR was reported to be $2\%$ in January 2020 [33], we consider a variation of $\pm 1\%$ around that value as reasonable for categorizing this epidemiological variable as: low if CFR $<1$, medium if $1\le$ CFR $<2$, high if $2\le$ CFR $<3$ and very high if CFR $\ge 3$.

**Table 3:** Estimated epidemiological typologies of 2020 and 2022 data

Variable	Typologies 2020				Typologies 2022			
	$\Gamma _{1}$	$\Gamma _{2}$	$\Gamma _{3}$	$\Gamma _{4}$	$\Psi _{1}$	$\Psi _{2}$	$\Psi _{3}$	$\Psi _{4}$
TNC (per million)	8399.88	71202.63	66895.50	29400.50	302028.76	127633.01	376966.28	144593.31
TND (per million)	89.82	766.22	1101.02	1121.34	864.75	369.78	2847.53	4926.29
TT (per thousand)	489.40	2579.38	384.08	160.10	25840.29	1732.06	1424.83	1180.78
CFR	0.95	1.11	1.67	3.75	0.29	0.31	0.76	3.37

Following this categorization, in 2020, $\Gamma _{1}$ is the only typology characterized by low case fatality rate, CFR $=0.95$. Even though the typologies $\Gamma _{1}$ and $\Gamma _{2}$ are very similar in CFR (0.95 and 1.11, respectively) they differ in TNC per million; typology $\Gamma _{1}$ represents countries with lower TNC (8399.88 per million) while typology $\Gamma _{2}$ is on the opposite side with 71202.63 TNC per million. Although typology $\Gamma _{4}$ has the highest of CFR (3.75), it has the second lowest value of TNC (29400.5 per million). Only TND per million closely resembles the pattern of CFR across typologies, i.e., the typologies with lowest values of CFR rates are also the ones with lower TND per million ($\Gamma _{1}$ and $\Gamma _{2}$). Testing was and still is regarded by some countries as the central method to control COVID-19 [28]. Throughout the pandemic, testing has steadily increased in line with lab capacity and the availability of lateral flow devices. As a result, the more tests carried out, the greater the likelihood of more positive cases being found [8]. Our typologies do not fully support this argument since we have typology $\Gamma _{2}$ and $\Gamma _{3}$ with similar values of TNC per million (71202.63 and 66895.50, respectively) but very different figures for TT per thousand (2579.38 and 384.08, respectively).

In 2022, there are three typologies with a low case fatality rate (i.e., CFR $<1$), namely $\Psi _{1}$, $\Psi _{2}$, and $\Psi _{3}$. Despite this common feature, the behavior of the remaining epidemiological variables is diverse across these typologies: typology $\Psi _{1}$ has the highest value for TT (25840.29) while $\Psi _{2}$ has a much lower value (1424.83); TND is 2847.53 in typology $\Psi _{3}$ but much lower in typology $\Psi _{2}$ (369.78). In this year, only $\Psi _{4}$ typology has a CFR above 3 and comes associated with the lowest TT and with the highest TND per million. This heterogeneity in the profiles is a likely consequence of different strategies to prevent and control transmission but may also be tied to indicators such as GDP and GHS [27] that differ across the countries under analysis. Table 4 presents the membership degree $\mu _{ik}$ in each typology for every country, regarding 2020 data.

**Table 4:** Countries’ membership degree in each typology in 2020

	Membership in Typology				Estimated
Country	$\Gamma _{1}$	$\Gamma _{2}$	$\Gamma _{3}$	$\Gamma _{4}$	CFR
Austria	0.38	0.02	0.43	0.17	1.75
Belgium	0.00	0.07	0.43	0.50	2.67
Bulgaria	0.00	0.00	0.00	1.00	3.74
Croatia	0.14	0.00	0.67	0.19	1.95
Cyprus	0.72	0.28	0.00	0.00	0.99
Czech Republic	0.01	0.00	0.99	0.00	1.66
Denmark	0.54	0.46	0.00	0.00	1.02
Estonia	0.83	0.02	0.15	0.00	1.06
Finland	0.86	0.00	0.00	0.14	1.35
France	0.15	0.10	0.26	0.48	2.51
Germany	0.61	0.03	0.03	0.32	1.88
Greece	0.35	0.00	0.00	0.65	2.77
Hungary	0.13	0.00	0.19	0.68	2.99
Ireland	0.53	0.02	0.00	0.45	2.21
Italy	0.00	0.10	0.01	0.89	3.46
Latvia	0.68	0.03	0.10	0.18	1.54
Lithuania	0.30	0.10	0.60	0.00	1.40
Luxembourg	0.00	1.00	0.00	0.00	1.11
Malta	0.55	0.24	0.00	0.21	1.59
Netherlands	0.37	0.00	0.61	0.02	1.45
Norway	1.00	0.00	0.00	0.00	0.95
Poland	0.24	0.00	0.32	0.44	2.43
Portugal	0.37	0.08	0.39	0.16	1.69
Romania	0.26	0.00	0.24	0.50	2.53
Slovakia	0.43	0.12	0.45	0.00	1.29
Slovenia	0.00	0.00	0.75	0.25	2.19
Spain	0.06	0.10	0.28	0.57	2.76
Sweden	0.34	0.03	0.42	0.21	1.84
Switzerland	0.18	0.00	0.70	0.12	1.80
United Kingdom	0.04	0.22	0.01	0.73	3.03

Countries like Norway, Luxemburg, Czech Republic, and Bulgaria show full or strong agreement ($\mu _{ik}$ equal or close to 1) with the unveiled typologies, i.e. with $\Gamma _{1}$, $\Gamma _{2}$, $\Gamma _{3}$ and $\Gamma _{4}$, respectively. We recall that these typologies are arranged in increasing order of CFR and $\Gamma _{1}$ is the only one characterized by a low CFR in 2020. On the opposite side, we find a fuzzy behavior in countries like Austria, France, Poland, Portugal, Slovakia, and Sweden, notably, because no membership degree is higher than 0.5 in any typology. It is also worth noting the cases of Germany, Ireland and Greece which suggest the existence of two extreme subpopulations, considering their predominant membership in $\Gamma _{1}$ and $\Gamma _{4}$. Table 4 also includes the estimated CFR^3^ for every country *k*, calculated using the formula

7 $$\begin{aligned} \text {CFR}\left( k\right) =\sum \limits _{i=1}^{4}\mu _{ik}\Gamma _{i},k=\text {Austria, ...,UK,} \end{aligned}$$

where the values $\Gamma _{i}$ are given in Table 3.

**Fig. 7:**
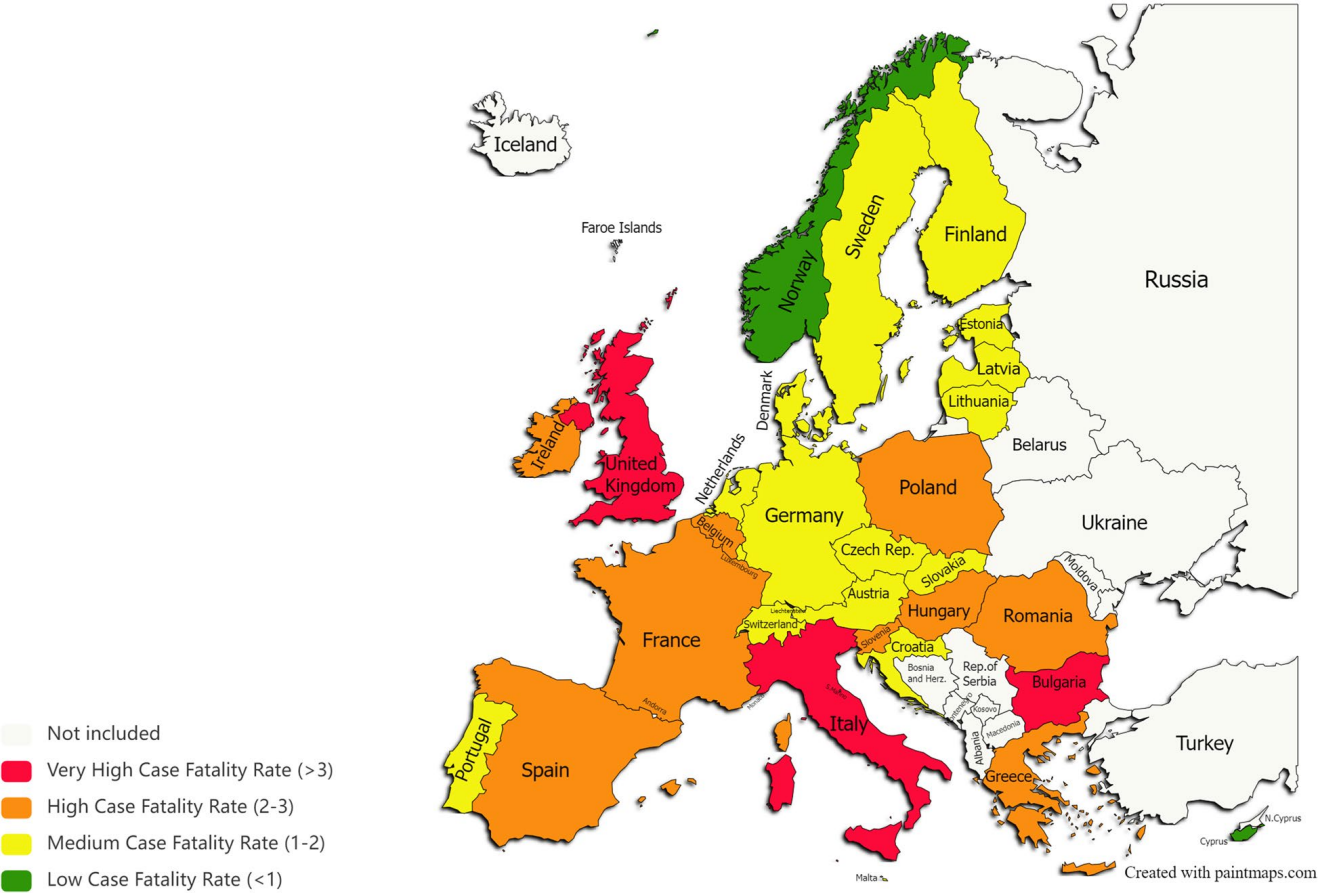
Countries grouped according to the estimated case fatality rate in December 2020 (graphic software from https://paintmaps.com/map-charts/71/Europe-map-chart)

We used the same categorization as above to cluster countries by the estimated CFR and mapped them with the following colors: green $\left( \text {CFR}<1\right)$, yellow $\left( 1\le \text {CFR}<2\right)$, orange $\left( 2\le \text {CFR}<3\right)$ and red $\left( \text {CFR}>3\right)$. This led us to represent a pictorial distribution of CFR due to COVID-19 in Europe by December, 2020 (Fig. 7). Clearly, in 2020 the pandemic situation across Europe was out of control, since most countries are classified as having medium or high case fatality rate, and three, Bulgaria, Italy and UK are in the red group. The green group comprises only Norway and Cyprus.

**Fig. 8:**
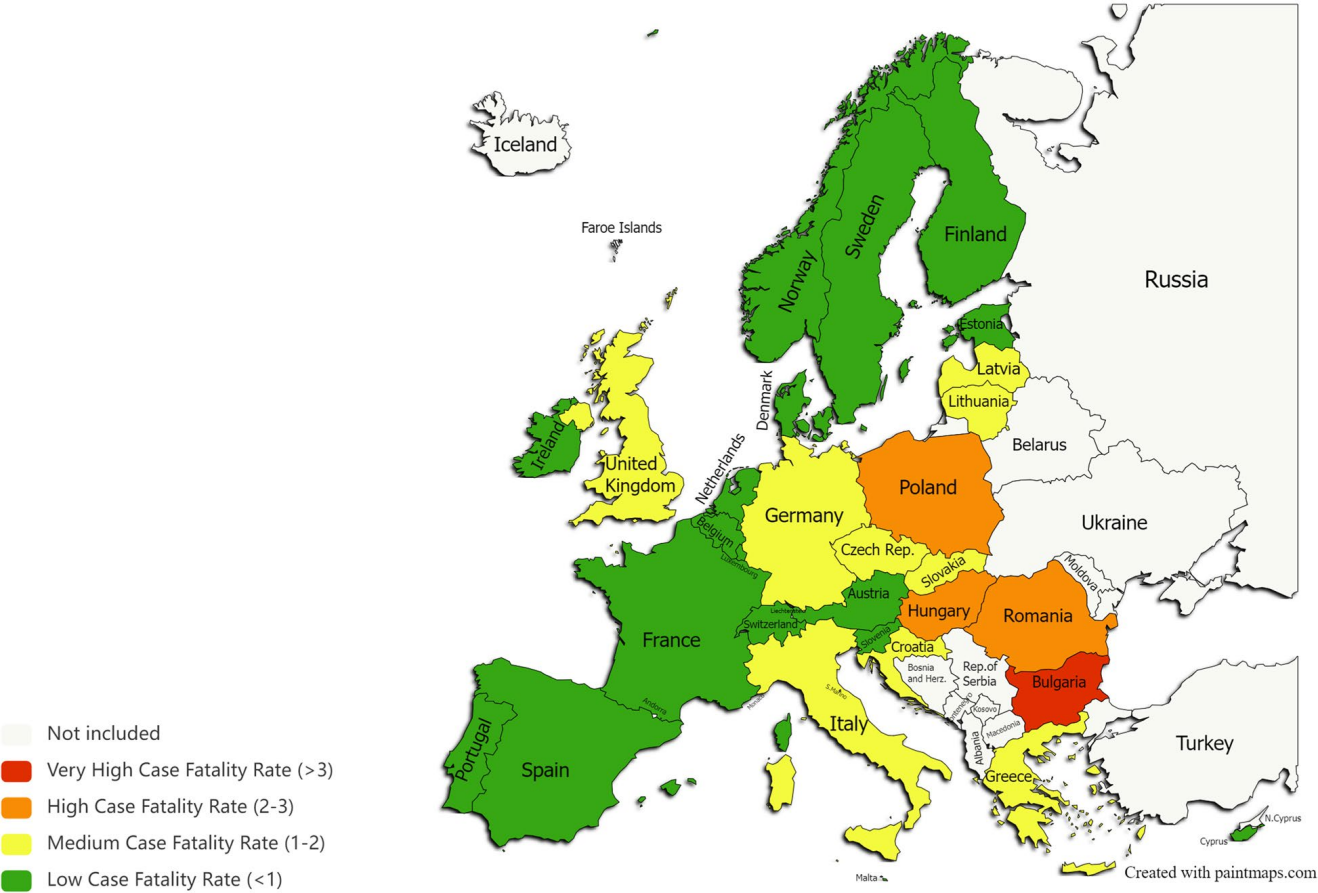
Countries grouped according to the estimated case fatality rate in February 2022 (graphic software from https://paintmaps.com/map-charts/71/Europe-map-chart)

Following the same procedure for 2022 data, Table 5 presents the membership degrees $\mu _{ik}$ in each typology $\Psi _{i}$ and the estimated CFR^4^ according to the equation (7), for every country. Figure 8 shows an updated map of Europe regarding the distribution of COVID-19 severity as assessed by CRF.

**Table 5:** Countries’ membership degree in each typology in 2022

	Membership in Typology				Estimated
Country	$\Psi _{1}$	$\Psi _{2}$	$\Psi _{3}$	$\Psi _{4}$	CFR
Austria	0.60	0.23	0.00	0.17	0.83
Belgium	0.05	0.23	0.58	0.13	0.97
Bulgaria	0.00	0.00	0.00	1.00	3.37
Croatia	0.00	0.17	0.46	0.37	1.65
Cyprus	1.00	0.00	0.00	0.00	0.29
Czech Republic	0.12	0.00	0.54	0.34	1.60
Denmark	0.42	0.18	0.40	0.00	0.48
Estonia	0.04	0.39	0.58	0.00	0.57
Finland	0.00	0.97	0.00	0.03	0.39
France	0.09	0.25	0.64	0.02	0.66
Germany	0.00	0.75	0.00	0.25	1.08
Greece	0.16	0.37	0.16	0.30	1.31
Hungary	0.00	0.10	0.14	0.76	2.69
Ireland	0.03	0.55	0.42	0.00	0.50
Italy	0.06	0.40	0.20	0.33	1.41
Latvia	0.07	0.28	0.40	0.25	1.24
Lithuania	0.06	0.16	0.57	0.21	1.22
Luxembourg	0.20	0.38	0.39	0.03	0.57
Malta	0.06	0.77	0.00	0.17	0.83
Netherlands	0.03	0.44	0.54	0.00	0.55
Norway	0.03	0.90	0.08	0.00	0.34
Poland	0.00	0.44	0.00	0.56	2.02
Portugal	0.09	0.29	0.56	0.06	0.74
Romania	0.00	0.33	0.00	0.67	2.37
Slovakia	0.28	0.00	0.49	0.23	1.23
Slovenia	0.00	0.00	0.99	0.01	0.78
Spain	0.01	0.47	0.36	0.16	0.97
Sweden	0.01	0.55	0.37	0.08	0.72
Switzerland	0.03	0.43	0.53	0.00	0.55
United Kingdom	0.21	0.24	0.38	0.17	1.01

Comparing Figs. 7 and 8, there is a noticeable significant change in the epidemiological situation from 2020 to 2022. Globally, 17 out of 30 countries reached a low case fatality rate (CFR $<1$) in 2022 while in 2020 only two countries were in this condition. Some significant outcomes are worth highlighting. First, only Bulgaria remained in red (CFR $>3$), a status that did not change from 2020. Poland, Hungary, and Romania are another set of countries that have not progressed favorably from 2020 to 2022, remaining unchanged in terms of color, orange. Secondly, Norway and Cyprus were the countries performing best during the pandemic crisis since they were able to maintain their green status during the 14-month period under analysis. Additionally, countries like Germany, Czech Republic, Slovakia, Latvia, Lithuania and Croatia consistently kept their initial yellow status meaning that they could control the pandemic quite effectively. Finally, the United Kingdom and Italy saw a very positive evolution since they migrated from red to yellow in the period under analysis. Overall, all these outcomes reveal a great improvement in the pandemic control in most European countries.

### Determinants of Case Fatality Rate

We used the MLR model (6) to determine how the four factors, vaccination (VAC), aging (AGE65), booster dose (BD) and stringency (ST) affect the case fatality rate (CFR). Using the original variables, we realized that these factors explain $58\%$ $\left( R_{adj}^{2}=0.58\right)$ of the variation in the target variable, i.e. in CFR. However, a convenient transformation of the variables involved in this analysis can considerably improve the relationship between them. Specifically, exponentiating CFR and using a log transformation of every independent variable (factor), i.e. using the MLR model

8 $$\begin{aligned} \exp \left( \text {CFR}\right) =B_{0}+B_{1}\times \ln \left( \text {VAC}\right) +B_{2}\times \ln \left( \text {AGE65}\right) +B_{3}\times \ln \left( \text {BD}\right) +B_{4}\times \ln \left( \text {ST}\right) +\varepsilon \end{aligned}$$

increases the explanation to $68\%$ $\left( R_{adj}^{2}=0.68\right)$. Table 6 displays the estimates of model (8) parameters. We realize that the coefficients $B_{3}$ and $B_{4}$ are not significant $\left( p>0.05\right)$, meaning that we found no empirical evidence on the importance of the booster dose and stringency over the fatality rate. We also note that $B_{2}$ is only significant at 0.1, meaning that the percentage of older people does not strongly impact countries’ CFR. Primary vaccination is the only independent variable with a significant impact on CFR $\left( p<0.01\right)$. Specifically, it affects the target variable five times more than does age, as measured by the respective beta coefficients, $-1.0$ vis-à-vis 0.2 (Table 6). We must stress that the non-significant effect of the booster dose can be a consequence of a non-negligible multicollinearity in model (8). To be more precise, the variance inflation factor (VIF) estimated for this variable was 6.8 and it was 6.4 for the primary vaccination, a little higher than the consensual upper bound of 5, in both cases. Nonetheless, this might have a moderate-to-weak impact on CFR. In fact, by removing the booster variable from model (8), the explanatory capacity of the reduced model still remains at $68\%$ and primary vaccination maintains its significant and strong effect on mortality reduction^5^.

**Table 6:** Unstandardized (*B*) and standardized (beta) estimates of the coefficients of the MLRM (8)

Model	*B*	Std. Error	Beta Coef.	t	Sig.
Constant ($B_{0}$)	62.4	26.7		2.3	0.03
VAC ($B_{1}$)	$-25.6$	6.5	$-1.0$	$-3.9$	$<0.01$
AGE65 ($B_{2}$)	9.5	4.7	0.2	2.0	0.06
BD ($B_{3}$)	2.5	3.1	0.2	0.8	0.43
ST ($B_{4}$)	3.2	4.7	0.1	0.7	0.50

**Fig. 9:**
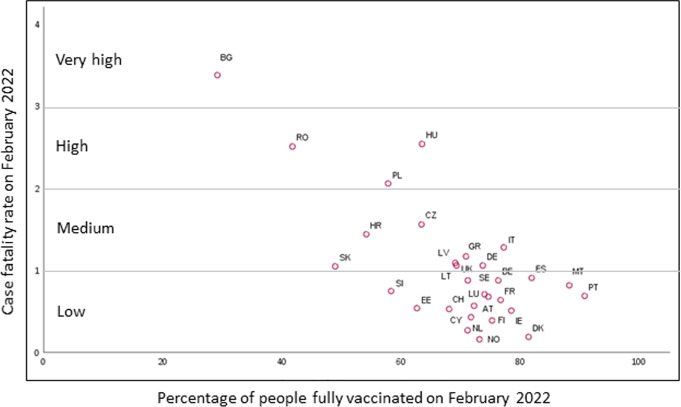
Scatter plot of case fatality rate and percentage of fully vaccinated people

Figure 9 shows how CFR relates to the vaccination rate in the 30 countries under analysis. It is evident that countries with a higher immunization rate exhibit lower CFR (e.g., Denmark and Portugal), which corroborates with the estimated regression model (8). Bulgaria is the country with worst situation with CFR $>3$ and the lowest percentage of fully vaccinated people. We also note that most countries reached the primary vaccination rate above $70\%$ by February 2022, and cluster under small values of CFR (below 1). Despite the heterogeneity among countries, in terms of population, geographical location or even the strategy adopted to face the pandemic, mass vaccination seems to be crucial to dramatically reduce CFR.

## Discussion

This study described the COVID-19 prevalence one year after the beginning of the pandemic (about December 2020) and one year after beginning of mass vaccination (about February 2022) in 30 European countries. The total number of deaths (per million inhabitants) was the variable that most differentiated the countries in 2020 (see Table 1), thus revealing that during the first year the countries were affected by the epidemic at different magnitudes. By 31 December 2020, Belgium was the country registering the highest number of deaths, 1678.7 per million, in contrast to Norway where fewer than 80 deaths per million were observed. By February 2022, the variable with greatest variability among countries was the total number of tests; whereas Poland was conducting 817.2 tests per thousand inhabitants, Cyprus reached 26077.7 tests per thousand. This suggests that countries undertook different strategies to prevent and control the pandemic.

The $\Gamma _{2}$, $\Gamma _{3}$, and $\Gamma _{4}$ typologies (2020) include countries where the pandemic has had the greatest impact (CFR $>1$), with the latter typology reaching a severe situation of case fatality rate above the global average of 2 percent reported by WHO [33]. These typologies were concomitantly associated with a high number of cases (per million) and a high number of deaths (per million). However, we found some possible inconsistency in emerged profiles: $\Gamma _{4}$ typology, that includes countries with the highest case fatality rate (CFR $=$ 3.75), did not register the highest total number of cases, and even the total number of deaths is only slightly higher than that in typology $\Gamma _{3}$, where the case fatality rate is much lower (CFR $=1.67$). Most probably the inconsistency is caused by the specificity of each epidemiological indicator. While total number of cases and total number of deaths are computed as ratios over the country’s total population, case fatality rate is computed over the total number of confirmed cases of COVID-19 within the country. This latter indicator is therefore more reliable to describe mortality by COVID-19 than total number of deaths. By February 2022, about one year after the beginning of mass vaccination, a strong improvement is found in country status: only one typology, i.e. $\Psi _{4}$, exhibits a very high case fatality rate (3.37). This typology also registers the lowest number of total tests per hundred and the highest number of total deaths per million. Among the remaining typologies, all characterized by a case fatality rate below one, there is great variability in the total number of deaths and total number of tests. On one hand, this reveals that the strategies undertaken by the countries to control the spread of the virus and the impact of COVID-19 on mortality were not uniform but, in the end, a similar outcome was achieved, as seen by a significant reduction in mortality. On the other hand, it strengthens the idea that the total number of cases per million and the total number of deaths per million are not the most feasible indicators to monitor the pandemic; case fatality rate is instead a more reliable metric, because it takes into consideration solely the confirmed cases of COVID-19.

When evaluating the association of case fatality rate with non-epidemiological variables, the outcomes reveal that primary vaccination (two doses) seems to be the most important to reduce case fatality rate. It is no coincidence that the countries classified as orange (Hungary, Poland, Romania) or red (Bulgaria) are the ones with lowest percentage of primary vaccination; by February 2022 these countries had less than $65\%$ of their population fully vaccinated and Romania and Bulgaria did not reach $50\%$. The non-significant effect of the booster variable does not entail the irrelevance of administrating booster shots. It instead suggests that providing everyone with primary vaccination can be more effective in reducing case fatality rate than alternatively proceeding with the administration of a third or even a fourth dose to people already vaccinated. Finally, the stringency index did not have a significant impact on reducing case fatality rate. Even so, the ECDC [9] recommends countries to resort to this kind of precautionary measures until there is worldwide coverage of effective vaccination.

## Strengths and limitations

The fuzzy approach to data analysis in one of the major strengths of this study. The underlying possibility of positioning countries in a structure set out by 4-cluster typologies, rather than forcing them to a mutually exclusive classification, helped understand how differently they responded to the pandemic. However, different approaches do not seem to substantially influence the case fatality rate (CFR) and, according to our study, it is a more reliable factor to account for the severity of COVID-19. In subsequent analysis, we also noticed a significant CFR reduction with (only) primary vaccination, i.e. two doses.

Several limitations of our study are worth noting, beginning with the stringency index. Despite the non-significant effect of this factor on CFR, it does not necessarilly entail its irrelevance in controlling the pandemic. We note that the stringency index is a composite indicator and it changed during the pandemic; however, we did not take into account its fluctuations when conducting a MLR analysis, and instead used its average value by February 2022. This can explain why it appears as a non-significant factor in predicting the CFR. Additionally, stringency measures, like stay-at-home requirements, restrictions on public gatherings, schools and workplaces closures, were stronger during the first year of the pandemic and countries progressively reduced these restrictions after starting vaccination.

Our study could not account for the variants of COVID-19 virus and their eventual effect on CFR. In fact, the mortality data reported in daily basis by OWD include the total number of deaths and the case fatality rate but do not provide information on the associated COVID-19 variant. As another limitation, the study was conducted solely in Europe which, despite the differences found among countries, is one of the geographical regions with medium-to-good pandemic control. We therefore think that it cannot directly be extrapolated to countries with distinct epidemic and health care conditions. However, it provides basis for similar studies in other regions of the world as well as for a more local or intra-country analysis in addition to that carried out in France [20] or in the USA [31].

## Conclusions

This study revealed four epidemiological typologies for both periods under analysis (end of 2020 and beginning of 2022). The clearest sign of change in the two periods concerns the case fatality rate that is found to be low in a single typology in 2020 but occurs in three typologies in 2022, although to different degrees. Among the factors we studied in this paper, i.e. primary vaccination, age ($65+$), booster vaccination and stringency measures, as determinants of case fatality rate by COVID-19, only primary vaccination had a significant positive impact on mortality reduction. This outcome highlights the importance of massive vaccination worldwide. WHO [36] established “achieving of $70\%$ coverage with COVID-19 vaccines in all countries by mid-2022 as a global imperative”, but many countries have failed to meet this goal for different reasons. Moreover, the Independent Allocation of Vaccines Group’s review of COVAX alerts for circumstances that can compromise that goal, namely the possible need for variant-specific vaccines, changes to vaccination policies, country preference for some products over others, the programmatic complexity of managing multiple products, and the need for better intelligence on country level planning and execution [36]. Despite its favorable evolution, the end of the pandemic situation has not been yet declared by WHO. Therefore, the academia must continue to pursue its key role in helping understand the past and the present and to deepen a prospective analysis of COVID-19 prevalence.

## Supplementary Information

**Additional file 1.**

## Data Availability

Data used in this analysis is available for open public access at https://github.com/owid/COVID-19-data/tree/master/public/data. Individual data were not used.

## Footnotes

1 In-depth details of the index can be found at: https://ourworldindata.org/metrics-explained-COVID19-stringency-index.

2 Readers unfamiliar with the fuzzy sets theory may wish to look at the Supplementary File where we provide a brief account on it.

3 The Person’s correlation coefficient between the estimated and the observed CFR is $\hat{\rho }=0.97$ $\left( p<0.001\right)$, which supports the reliability of our approach.

4 The Person’s correlation coefficient between the estimated and the observed CFR is in this case $\hat{\rho }=0.99$ $\left( p<0.001\right)$.

5 Here, VIF $\simeq 1$ for all three remaining factors.
