# Supplementary material for: COVID-19 in Europe: from outbreak to vaccination
Source: BMC Public Health. 2022 Dec 2;22:2245. doi: 10.1186/s12889-022-14454-5 (PMC9716177; doi:10.1186/s12889-022-14454-5)
Supplement: Supplementary file 1 — Additional file 1. [file 12889_2022_14454_MOESM1_ESM.pdf]

A brief note on fuzzy sets

Given a non-empty set $\mathbf{\Omega} \subseteq \mathbb{R}^n$, a fuzzy set A in $\mathbf{\Omega}$ is a set of ordered pairs [1]

$$A = \{(\mathbf{x}; \mu_A(\mathbf{x})), \quad \mathbf{x} \in \mathbf{\Omega}\}$$

where $\mu_A(\mathbf{x}) \in [0,1]$ is the membership degree of $\mathbf{x} \in \mathbf{\Omega}$ in A. We use the notation $A \leftrightarrow \mu_A$ to highlight the association between a fuzzy set and the corresponding membership function. If $\mu_A(\mathbf{x}) = 1$, $\mathbf{x}$ is a full member of A in the classical sense, and may be referred to as prototype of this set. So, prototypes are useful to identify or label fuzzy sets.

We say that the $c \geq 2$ fuzzy sets $A_1 \leftrightarrow \mu_{A_1}$, $A_2 \leftrightarrow \mu_{A_2}$, ..., $A_c \leftrightarrow \mu_{A_c}$ in $\mathbf{\Omega}$ form a fuzzy c-partition of $\mathbf{\Omega}$ if, for $i = 1, 2, ..c$, A_i has at least one prototype and

$$\forall \mathbf{x} \in \mathbf{\Omega}, \ \sum_{i=1}^{c} \mu_{A_i}(\mathbf{x}) = 1,$$

(see [2]). Therefore, each element $\mathbf{x} \in \mathbf{\Omega}$ is represented in a fuzzy c-partition by the unit sum vector

$$\boldsymbol{\mu}(\mathbf{x}) = \left(\mu_{A_1}(\mathbf{x}), \mu_{A_2}(\mathbf{x}), ..., \mu_{A_c}(\mathbf{x})\right), \tag{1}$$

where its ith coordinate accounts for the degree of belongingness of $\mathbf{x}$ in A_i.

In general, a fuzzy analysis aims to estimate a fuzzy c-partition that hypothetically underlies the universe $\mathbf{\Omega}$ from where the observed data $\mathbf{X}$ are sampled. Since $\mathbf{X}$ is a countable subset of $\mathbf{\Omega}$, we can alternatively write (1) for the kth observation as follows:

$$\boldsymbol{\mu}_k = (\mu_{1k}, \mu_{2k}, ..., \mu_{ck}), \quad k = 1, 2, .., N, \tag{2}$$

where N is the sample size. A fuzzy analysis therefore entails the estimation of the prototypes of every fuzzy c-partition set and, for each observation, the respective membership degrees vector as in (2). Usually, the prototypes are

arranged in a matrix in columns, e.g., $\mathbf{V} = [v_{ji}] \in \mathbb{R}^{n \times c}$, and the membership degrees are grouped in the so-called partition matrix, $\mathbf{U} = [\mu_{ik}] \in [0, 1]^{c \times N}$. In this perspective, the outcome of a fuzzy approach to data analysis are the estimates of $\mathbf{U}$ and $\mathbf{V}$, given $\mathbf{X}$.

References

[1] Bellman, R.E., Zadeh, L.A (1970). Decision-making in Fuzzy Environment, Management Science, 17(4), B141–B164.

[2] Nguyen, H.T., Prasad, N.R., Walker, C.L., Wlaker, E.A. (2003). *A First Course in Fuzzy and Neural Control*. Chapman & Hall/CRC.
